# Effects of glycolysis and polyamine predation on intestinal epithelial barrier in colorectal cancer

**DOI:** 10.3389/fonc.2022.961257

**Published:** 2022-07-15

**Authors:** Yu Wang, Huan He, Jingwen Chen, Zijing Song, Xuediao Pan, Tian Lan, Guixiang Wang

**Affiliations:** School of Pharmacy, Guangdong Pharmaceutical University, Guangzhou, China

**Keywords:** colorectal cancer, polyamines, HIF1α, MYC, intestinal permeability

## Abstract

Colorectal cancer (CRC) is the second most lethal cancer and the third most common cancer in the world, and its prognosis is severely affected by high intestinal mucosal permeability and increasing tumor burden. Studies have shown that the expression of hypoxia induce factor 1α (HIF1α) is up-regulated in a variety of tumor tissues, which is related to multiple metabolic reprogramming of tumor cells. However, the role of HIF1α in CRC tumor growth, tumor polyamine metabolism and intestinal mucosal barrier damage has not been studied. Here, we constructed different types of CRC tumor-bearing mice models by inoculating HCT116 cells with different levels of HIF1α expression (knockdown, wild type, overexpression) in the armpits of mice to explore the upstream and downstream regulators of HIF1α, the effects of HIF1α on the growth of CRC, and the CRC polyamine metabolism and its effect on the intestinal mucosal barrier. We found that with the increase of HIF1 gene expression, tumor growth was promoted and intestinal mucosal permeability was increased. The expression of glycolysis-related proteins was up-regulated, the rate-limiting enzyme ODC of polyamine synthesis was decreased, and the transfer protein of polyamine was increased. HPLC showed that the polyamine content in the tumor tissue of the overexpression group HIF1α OE was higher than that of the wild group HIF1α (+/+), and higher than that of the knockdown group HIF1α (-/-), but the content of polyamines in intestinal mucosa was the opposite. After supplementation of exogenous polyamines, the content of polyamines in intestinal mucosa and tumor tissue increased, and the damage of intestinal mucosa was alleviated. In conclusion, upon activation of the MYC/HIF1 pathway, tumor glycolysis is enhanced, tumors require more energy and endogenous polyamine synthesis is reduced. Therefore, in order to meet its growth needs, tumor will rob polyamines in the intestinal mucosa, resulting in intestinal mucosal epithelial barrier dysfunction.

## 1 Introduction

Colorectal cancer (CRC) is one of the most common types of cancer in the world and a significant cause of cancer-related deaths (1). The incidence of CRC has steadily increased over the past decade. In the United States, the incidence of CRC among adults under the age of 50 increased by 22%, the mortality rate of CRC has increased by 13%, and the age of onset has been decreasing year by year (2, 3). According to the global cancer statistics in 2020, there are 555000 new cases of CRC in China, ranking the third among malignant tumors. The incidence of CRC is 23.9/100,000, and the mortality rate is 12.0/100,000, and the incidence rate of males is higher than that of females (4, 5). Genetic and environmental factors are closely related to the occurrence and development of CRC. The occurrence and development of CRC is caused by the accumulation of multi-step carcinogenic process influenced by lifestyle and dietary factors, while hereditary CRC accounts for only 5-10% of all CRC cases (6). With the in-depth study of the pathogenesis of CRC, it has been found that it involves multiple molecular pathways, especially genetic and epigenetic events (7, 8).

Polyamines are a class of low molecular aliphatic cationic compounds widely existing in eukaryotic cells, mainly including putrescine, spermine and spermidine (9). They play a key role in vital life activities such as cell proliferation, differentiation and embryonic development (10). Studies have found that abnormal polyamines are closely related to the malignant process of tumors, and the levels of polyamines in the urine and blood of cancer patients will be abnormally increased. Therefore, the content of polyamines in the urine and blood of tumor patients have been used as an important clinical reference to determine the prognosis of tumors (11, 12). The levels of polyamines in tumor cells are precisely regulated by polyamine synthase, catabolic enzymes and their transmembrane transport system (13). The concentration of polyamines are significantly increased in the development and progression of epithelial-related cancers such as colon and skin cancers (14). The pro-proliferative effect of polyamines is necessary for tumor growth and is also important for maintaining the integrity of the intestinal mucosal barrier (15). Removal of polyamines using 2-difluoromethylornithine (DFMO: ODC inhibitor) which leads to down regulation of Occludin, Claudin and Zonula occludens (ZOs) expression in IEC-6 cells, and disturbance of epithelial barrier function, suggests that polyamines are necessary for the synthesis and stability of connexin (16, 17). Whereas polyamines are closely related to tumor growth and maintaining the normal function of intestinal mucosal barrier, we speculate that there may be a mechanism during the growth of colorectal cancer that allows tumor tissue to prey on polyamines in the intestinal mucosa, resulting in intestinal epithelial barrier dysfunction. The vast majority of malignant tumors grow rapidly, and the tumors are in a state of relative ischemia and hypoxia. Under hypoxic conditions, HIF1α will accumulate inside tumor cells (18, 19). The massive accumulation of HIF1α broadly activates the expression of downstream genes and alters the energy metabolism of cells, that is, tumor cells obtain energy through glycolysis under both aerobic and hypoxic conditions (20, 21).The expression of proto oncogene myc and hypoxia inducible factor increased in colorectal cancer and they are key transcription factors of tumor cell glycolysis (22). They regulate the expression of glycolytic enzymes including hexokinase 2 (HK2), pyruvate kinase M2 isoform (PKM2), lactate dehydrogenase A under normoxia and hypoxia, and promote the survival of cancer cells and the growth of cancer tissues (23, 24). Hypoxic microenvironment is an important feature of solid tumor tissues. The hypoxic environment leads to tumor metabolic reprogramming, resulting in adaptive changes such as diminished oxidative phosphorylation, and HIF1 is a major regulator of this metabolic reprogramming (25). The transcription factor active form of HIF1 is a heterodimer of HIF1α and HIF1β, and its activity is controlled by intracellular oxygen content. Studies have shown that MYC is involved in the regulation of HIF1α expression: the overexpression of MYC can significantly improve the stability of HIF1α protein in breast cancer cells under normoxia and hypoxia; through post-transcriptional regulation, the overexpression of c-Myc can increase the expression of HIF1α in colorectal cancer; inhibition the expression of c-Myc in glioblastoma multiforme cells can down-regulate HIF1α transcription and reduce glycolysis (26–28). MYC gene encodes MYC protein, which can bind with MAX to form heterodimer. When the heterodimer binds to the E-box region upstream of ornithine decarboxylase (ODC) gene promoter, it can upregulate the expression of ODC (29). At the same time, polyamines synthesized by ODC can stimulate the expression of MYC to form positive feedback regulation (30, 31). Since the pro-proliferation effect of polyamines can increase the oxygen consumption of cells, whether the regulation of HIF1α expression by MYC is related to the upregulation of ODC by MYC and thus increases oxygen consumption by tumor cells.

**Figure 1:**
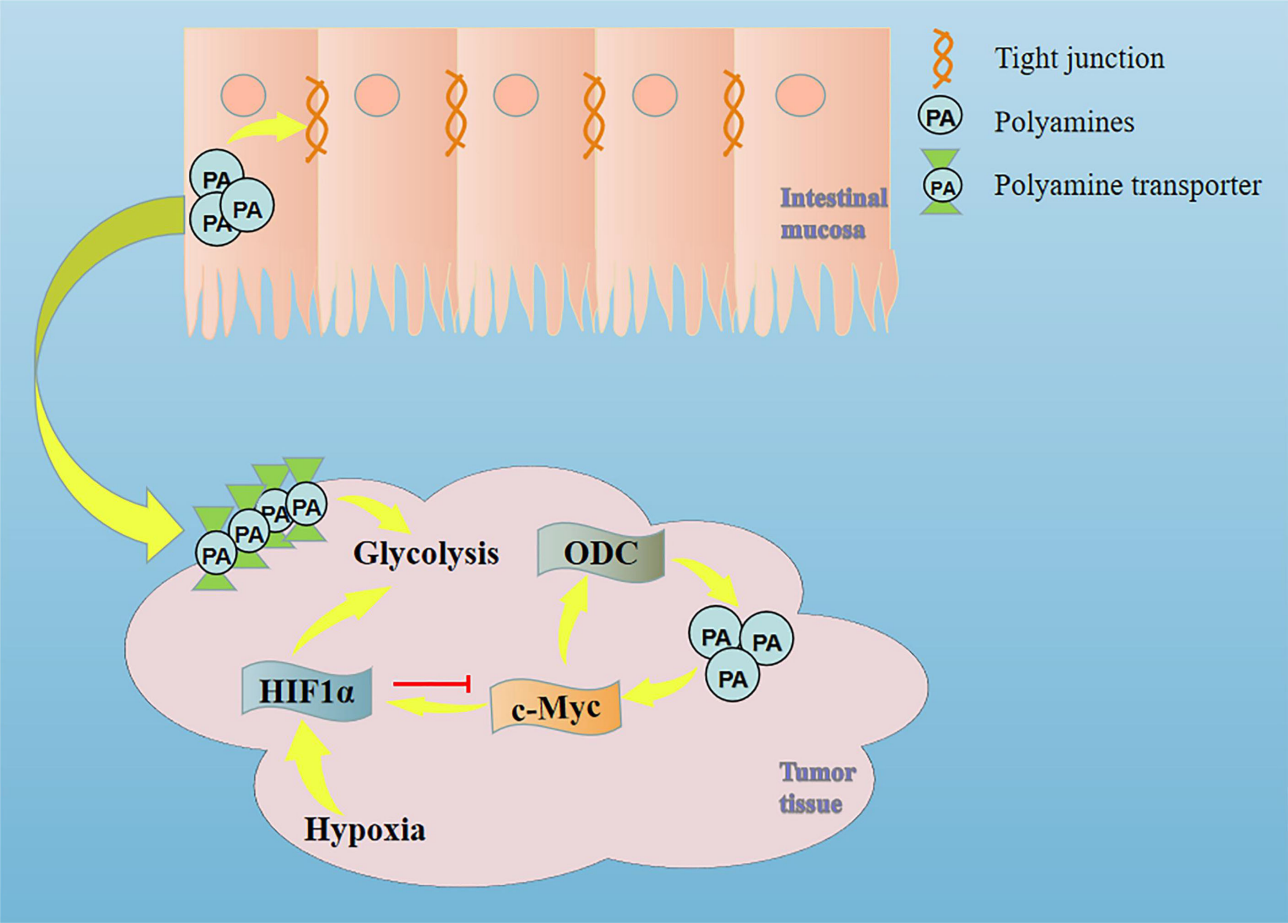
Mechanism of intestinal mucosal barrier damage caused by tumor tissue plundering polyamines in intestinal mucosal tissue.

However, when tumor cells are hypoxic, HIF1α can inhibit the transcription of ODC by binding to MAX and inducing the binding of MAX-interacting protein (MXI1) with MAX. The down-regulation of ODC (decreased intracellular polyamine synthesis) will increase the uptake of exogenous polyamines, which indicates that polyamines play an important role in the MYC/HIF1 pathway (32, 33). Given that c-Myc is a well-known oncogenic driver in CRC, Xiangjing Hu et al. provided strong pharmacological evidence to support the translation of dihydroartemisinin for the treatment of late-stage CRC by targeting c-Myc (34). Studies have shown that inhibiting the HIF1α pathway in CRC can play a role in suppressing tumors (35). Combined with our previous studies, berberine can deplete tumor polyamines and inhibit ODC expression, ultimately inhibiting tumor growth, reducing intestinal permeability in CRC patients, and downregulating MYC and HIF1α (36). Therefore, we speculate that in the deterioration of CRC, activation of MYC/HIF1 down-regulates the first rate-limiting enzyme in the polyamine synthesis pathway (ODC) and reduces the synthesis of endogenous polyamines, which makes the tumor tissue plunder the polyamines of intestinal mucosa to meet its metabolic needs (**Figure 1**). This may be an important reason for the dysfunction of intestinal mucosal barrier in patients with colorectal cancer.

## 2 Materials and methods

### 2.1 Materials

The research design and experimental technology were reviewed approved by the Institutional Review Committee, which is the Institutional Animal Care and Use Committee of Guangdong Pharmaceutical University. Purchase of SPF nude mice at Guangdong Animal Center (Certificate No. SYXK (Guangdong) 2017-0125). All animal handling and procedures follow the international guidelines for the use and care of laboratory research animals and comply with the regulations of the Institutional Animal Care. HCT116 cells were purchased from ATCC. Provided with technical support from Saiye Biological Co, Ltd, the HIF1α gene of HCT116 cell line was knocked out using CRISPR/Cas9 technology to obtain HIF1α (-/-) cell line, HCT116 cells were established by virus transduction technology to establish a stable transfected strain overexpressing HIF1α gene, and the obtained HIF1α OE cell line. The D-lactic acid detection kit was purchased from AmyJet Scientific Co.Ltd. Standards for putrescine, spermidine and spermidine were purchased from Pfifizer. Chromatographic grade methanol and acetonitrile were purchased from Opple. BCA kits were purchased from Thermo. PVDF membranes were purchased from Bio-Rad, USA. Primary antibodies for ODC, c-Myc and HIF1α were purchased from Abcam. Primary antibodies for ZO1, Occludin, spermidine/spermine N1 acetyltransferase (SSAT), OAZ1 and β-actin, and rabbit, mouse secondary antibodies were purchased from Proteintech. The remaining reagents were purchased from Guangzhou Chemical Reagent Factory.

### 2.2 Animal modeling and administration method

HCT116 cells with different expression levels of HIF1α were inoculated subcutaneously. When the tumor tissues were 10 mm × 10 mm, they were transplanted into the armpits of nude mice. According to the expression level of HIF1α, nude mice were divided into normal group, HIF1α (+/+) group, HIF1α OE group, HIF1α (-/-) group. Tumor-bearing mice with different expression levels of HIF1α were reared for four weeks and their body weights were recorded every other day. The body weight of tumor-bearing mice was found to decrease slightly or remain stable from around day 14, indicating that the intestinal mucosa of nude mice may be damaged at this time. The tumor-bearing mice in the HIF1α (+/+) group were gavaged spermidine (20mg/kg) for two weeks after inoculation for two weeks. During the administration period, the mental state, vital signs and food intake of nude mice were observed and recorded every day, and the tumor volume was observed and recorded every other day. After two weeks of gavage, the nude mice were sacrificed after 8 hours of fasting tumors were stripped and weighed. The small intestines of nude mice were collected and the intestinal mucosa was scraped. The tumor volume was calculated by the metric: V=0.5×a×b^2^ (a: tumor length; b: short tumor diameter).

### 2.3 Intestinal mucosal permeability test

Blood was collected from the eyeballs of nude mice in each group, and the blood was allowed to stand at room temperature for 30 min, and the supernatant was aspirated after low-temperature centrifugation. Serum D-lactic acid concentration of nude mice in each group was determined by Ai Meijie serum D-lactic acid kit. A standard curve was drawn based on the reaction between the D-lactic acid standard solution and the enzyme. In a 96-well plate, the prepared D-lactic acid working solution and serum samples were added, and incubated at room temperature for 1.5 h in the dark. The absorbance at 575nm and 605nm was measured by a microplate reader, and the ratio of OD575nm/OD605nm was calculated. Substitute into the standard curve to obtain the D-lactic acid concentration in the sample.

### 2.4 HE staining of intestinal mucosa and tumor tissue sections

When dissecting the tumor bearing mice, part of the tumor tissue and intestinal mucosal tissue were fixed in 4% paraformaldehyde for 24 hours, the fixed solution was washed off, and then the tissues were dehydrated in different concentrations of ethanol. Put the dehydrated tissue into xylene for transparency and paraffin with different melting points. Slice the embedded wax block and bake it in a 60°C constant temperature oven for 2h. Dehydrate and transparentize again, and finally drop an appropriate amount of neutral gum in the center of the slide, the morphological changes of different groups of tissues were observed by optical electron microscope.

### 2.5 Determination of polyamine content

(1) Put the tissue into a grinder for full grinding, quantitatively detect the protein content through BCA, adding 5% perchloric acid of 5 times the volume to the rest of the mixture, and take the supernatant after centrifugation for derivatization. (2) Add NaOH and benzoyl chloride to the tissue supernatant, add chloroform (shading) after full reaction, suck the lower organic layer after centrifugation, add ultra pure water, suck the lower solution after centrifugation, blow dry the extract with nitrogen, add 1mL acetonitrile to dissolve the residue and filter. (3) Chromatographic conditions: 20 μL for each loading, the chromatographic column is C18 column, the detection wavelength is at 229 nm, the mobile phase is acetonitrile and ultra pure water (38:62), and the flow rate is 1 ml/min,

### 2.6 Western blot

Determine the protein concentration of the sample by BCA method, prepare a gel of appropriate concentration, inject the protein sample into the gel hole, after electrophoresis, transfer the protein to PVDF membrane, block it with 5%BSA, incubate it with primary antibody at 4° covernight, incubate it with secondary antibody at room temperature for 1 hour the next day, wash the PVDF membrane with TBST, adding luminescent solution and put it into the imager for development, and analyze the gray value with software.

## 3 Results and discussion

### 3.1 Activation of the HIF1α pathway promotes tumor growth in tumor-bearing mice (HCT116)

**Figure 2:**
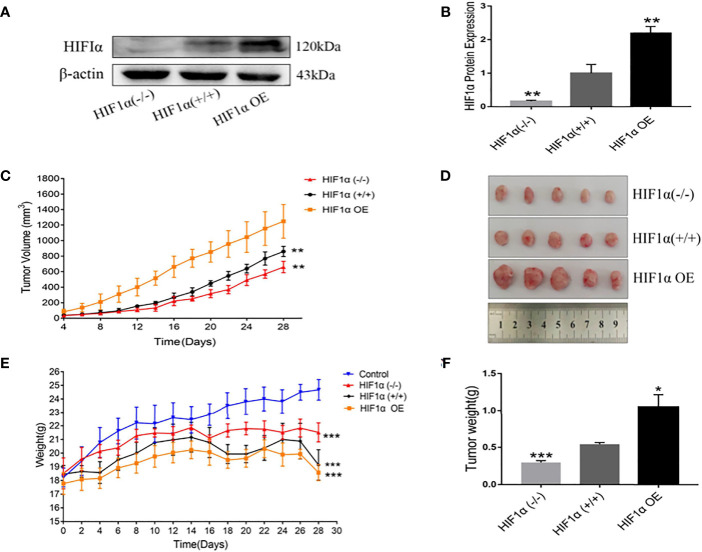
Upregulation of HIF1 can promote tumor growth. **(A)** Detection of HIF1 protein expression in tumor-bearing mice after construction of xenograft model. **(B)** Quantitative data of **(A)**. HIF1α (-/-) group, HIF1α OE group, ^**^ *P* < 0.01 *vs*. HIF1α (+/+) group. **(C)** Tumor volume changes in tumor- bearing mice during feeding (n =5). HIF1α (-/-) group, HIF1α OE group, ^**^ *P* < 0.01 *vs*. HIF1α (+/+) group. **(D)** Picture of xenograft tumor. **(E)** Changes in body weight of tumor-bearing mice during rearing. HIF1a group, ***P<0.001 compared with the control group. **(F)** Tumor tissue was dissected and weighed after four weeks of feeding. HIF1a (-/-) group, ***P < 0.001 vs. HIF1a (+/+) group; HIF1a OE group, *P < 0.5 vs. HIF1a (+/+) group.

HCT116 cell suspensions with different expression levels of HIF1α were injected subcutaneously into the armpits of nude mice to construct xenograft models. The expression of HIF1α protein in the tumors of HIF1 (+/+) group, HIF1α OE group and HIFα (-/-) group was verified by Western blot (**Figure 2A**), and the specific expression amount (**Figure 2B**). Compared with HIF1α (+/+) group, the expression of HIF1α protein in tumor tissue of HIF1α OE group was significantly increased. Although the HIF1α protein expression was significantly decreased in the tumor tissue of the HIF1α (-/-) group, there was still a small amount of expression. This indicates that the tumor-bearing mouse model was successful. During the rearing period, tumor major and minor diameters were measured by vernier calipers every other day. Compared with HIF1α (+/+) group, HIF1α (-/-) group had the slowest increase in tumor volume and HIF1α OE group had the fastest increase in tumor volume (**Figure 2C**). Four weeks later, tumors in the armpits of nude mice were dissected out and photographed (**Figure 2D**). As exhibited in **Figure 2E**, the weights of nude mice with different expression levels of HIF1α was lower than that of the normal group. The weight of HIF1α OE group is lower than that of the HIF1α (+/+) group, and the weight of the HIF1α (+/+) group is lower than that of the HIF1α (-/-) group. As shown in **Figure 2F**, the tumor mass of HIF1α OE group was larger than that of HIF1α (+/+) group and HIF1α (-/-) group. The above results suggest that the activation of the HIF1α pathway can promote tumor growth.

### 3.2 Activation of the HIF1α pathway can increase the damage of the intestinal mucosal barrier in tumor-bearing mice

**Figure 3:**
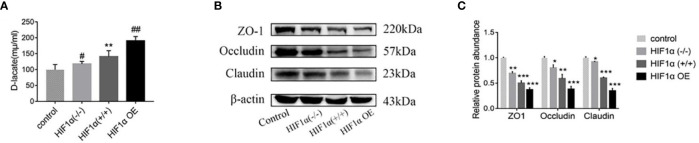
Upregulation of the HIF1α gene aggravates the permeability of the intestinal mucosa. **(A)** Blood was collected from the eyeball of nude mice after anesthesia, and the concentration of D-lactic acid in plasma was detected by ELISA (n = 5). HIF1α (+/+) group, ^**^ *P*<0.01 *vs*. control group; HIF1α (-/-) group, HIF1α OE group, ^#^ *P*<0.05, ^##^ *P*<0.01 *vs*. HIF1α (+/+) group. **(B)** The expression of ZO1 and occludin in the intestinal mucosa of tumor-bearing mice with different HIF1α gene expression was detected. **(C)** Quantitative data of **(B)**. HIF1α group, ^*^ *P*<0.05, ^**^ *P*<0.01, ^***^ *P*<0.001 *vs*. control group.

The differences in intestinal mucosal permeability of nude mice in each group are shown in **Figure 3**. When the intestinal mucosal barrier is damaged, D-lactic acid, one of the products of bacterial growth and metabolism in the gut, enters the circulating blood through the intestinal mucosal tissue. The higher the D-lactic acid concentration, the greater the permeability. The serum D-lactic acid concentration in the normal group was the lowest, and the serum D-lactic acid concentration in the tumor-bearing mice groups were higher than that in the normal group. The HIF1α OE group was higher than that of HIF1α (+/+) group, and that of HIF1α (+/+) group was higher than that of HIF1α (-/-) group. This indicates that the intestinal mucosa of the HIF1α OE group had the most severe intestinal mucosal damage and the greatest permeability (**Figure 3A**). Western blot was used to detect the expressions of tight junction proteins: ZO-1, Occludin and Claudin (**Figure 3B**). As shown in **Figure 3C**, the results showed that the tight junction proteins in the intestinal mucosa of tumor-bearing mice with three different HIF1α expression levels decreased in different degrees, and the degree of decline was: HIF1α OE group > HIF1α (+/+) group > HIF1α (-/-) group. Because the tight junction proteins are an important part of the first line of defense of the intestinal mucosal barrier, so this result indicates that with the up-regulation of HIF1α, the intestinal mucosal damage is progressively worsened.

**Figure 4:**
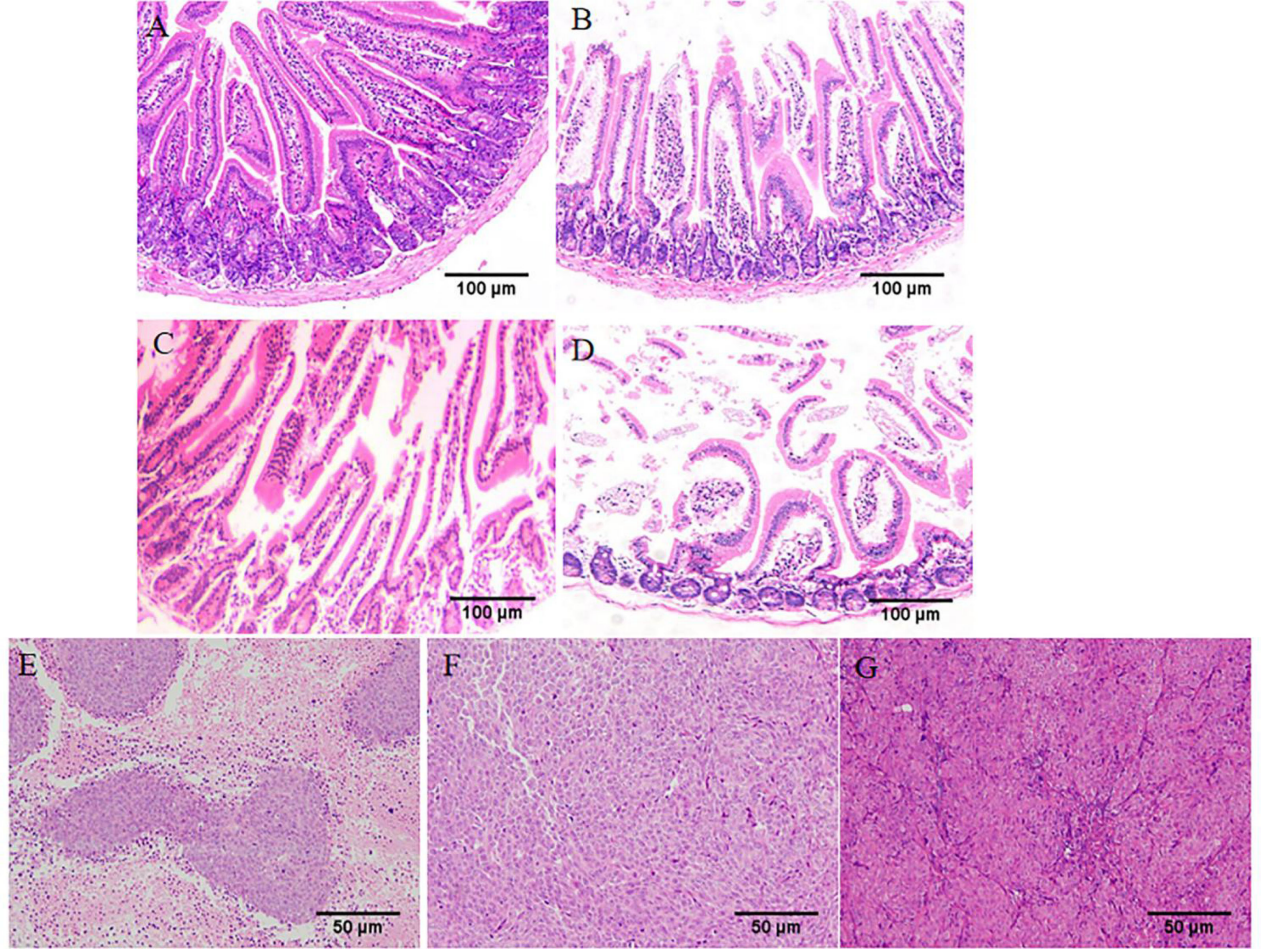
Upregulation of HIF1α can exacerbate intestinal mucosal destruction and increase tumor malignancy. Structural changes of intestinal mucosa of tumor-bearing mice were observed under light microscope (HE staining, ×100). **(A)** control group, **(B)** HIF1α (-/-) group, **(C)** HIF1α (+/+) group, **(D)** HIF1α OE group. The tumor structure was observed under a light microscope (HE staining, ×200). **(E)** HIF1α (-/-) group, **(F)** HIF1α (+/+) group, **(G)** HIF1α OE group.

Observed pathological sections under a 100x optical microscope. In the normal group, the intestinal mucosal glands were neatly arranged, erect, and close to the muscularis mucosae (**Figure 4A**). In the wild-type HIF1α (+/+) group, the villi were obviously broken, the crypt depth became deeper, and part of the central chyle duct disappeared. As shown in **Figures 4B–D**, Compared with the HIF1α (+/+) group, the glandular arrangement and integrity of HIF1α (-/-) group were improved, and the length of villi were increased; the glandular structure of HIF1α OE group was mostly disappeared, and the muscularis mucosa was thinned, the villi were shortened and the cells were disorganized. This indicates that the intestinal mucosal structural damage in tumor-bearing mice is aggravated after the activation of the HIF1 pathway. As shown in **Figures 4E–G**, observed under 200x microscope, tumor cells in HIF1α (+/+) group showed obvious atypia, with spindle-shaped tumor cells, rounded nuclei, and increased nuclear division. Compared with the HIF1α (+/+) group, the tumor parenchyma in the HIF1α (-/-) group was reduced. In the HIF1α OE group, the tumor parenchyma increased, the cell structure was blurred, the border was unclear, the nucleus was not obvious, and there were some tumor cells showed sheet necrosis.This may be because the tumor is too large, causing internal ischemia, hypoxia and necrosis.This may be caused by internal hypoxia-ischemia due to the large tumor.

### 3.3. Activation of the HIF1α pathway enables tumor tissue to prey on polyamines from intestinal mucosa to meet tumor glycolysis for more energy

**Figure 5:**
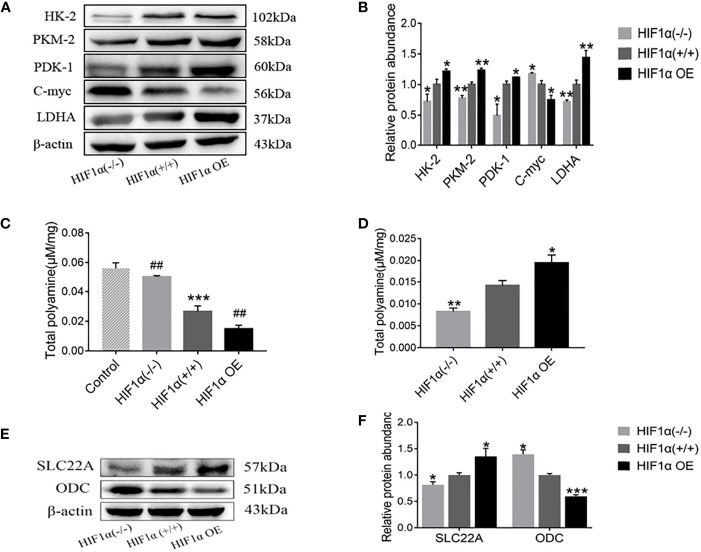
Activation of HIF1 promotes tumor glycolysis and promotes tumor deprivation of intestinal mucosal polyamines. **(A)** Detection of tumor glycolysis-related proteins in tumor-bearing mice with different levels of HIF1α expression. **(B)** Quantitative data of **(A)**. ber group, ^*^ *P* < 0.05, ^**^ *P* < 0.01 *vs*. HIF1α (+/+) group. **(C)** The total polyamine concentration in the intestinal mucosa was determined by HPLC. HIF1a (+/+) group, ***P < 0.001 vs. control group; HIF1a OE group, ##P < 0.01 vs. HIF1a (+/+) group; HIF1a (-/-) group, ##P < 0.001 vs. HIF1a (+/+) group. **(E)** Detection of tumor polyamine metabolism-related proteins in tumor-bearing mice with different levels of HIF1α expression. **(F)** Western blot quantitative data of **(E)**. ber group, ^*^ *P* < 0.05, ^***^ *P* < 0.001 *vs*. HIF1α (+/+) group.

The expressions of glycolysis-related enzymes (HK2, PKM2, PDK1 and LDHA) in HIF1α (-/-) group, HIF1α (+/+) group and HIF1α OE group were detected by Western blot (**Figure 5A**), and as shown in **Figure 5B**, the analysis showed that the gray value increased with the increase of HIF1α expression. These results indicate that the up-regulation of HIF1α expression can promote the glycolysis process of tumors. Since tumors obtain energy through glycolysis, up-regulation of HIF1α expression promotes tumor growth, which is also consistent with the results in 3.1.

In this paper, high-performance liquid chromatography was used to detect the content of polyamines in tissues. As exhibited in **Figure 5C**, the content of polyamines in intestinal mucosa of normal group was significantly higher than that of HIF1α(+/+) group. Compared with the HIF1α (+/+) group, the polyamine content in the HIF1α (-/-) group was increased, while the content in the HIF1α OE group was decreased. However, as showed in **Figure 5D**, the levels of polyamines in tumor tissue were opposite to those in intestinal mucosa: HIF1α OE group > HIF1α (+/+) group > HIF1α (-/-) group. The content of polyamines in tumor tissues with high expression of hif1 is high, and the content in intestinal mucosa tissue is low, while the expression of hif1 silence is the opposite. This indicates that the tumor preys on intestinal mucosal polyamines. As shown in **Figure 5E**, WB was further used to detect the expression levels of polyamine transporter SLC22A and polyamine synthesis rate-limiting enzyme ODC. The results of gray value analysis showed that with the up-regulation of HIF1α expression, the protein expressions of SLC22A increased (**Figure 5F**). SLC22A as a polyamine transfer protein, its increased expression suggests that overexpression of HIF1 promotes tumor transfer polyamines from outside. ODC is the rate-limiting enzyme for polyamine synthesis. The expressions of c-Myc and ODC decreased (**Figures 5A, F**), and the degree of decline is HIF1α OE group > HIF1α (+/+) group > HIF1α (-/-) group. This indicated that overexpression of HIF1α could inhibit the transcription of c-Myc target gene ODC. Decreased ODC reduces endogenous polyamine synthesis thereby promotes the predation of polyamines in the intestinal mucosa by the tumor to meet the tumor’s own growth needs.

### 3.4. Tumor tissue of tumor-bearing mice will not plunder intestinal mucosal polyamines after obtaining enough exogenous polyamines

**Figure 6:**
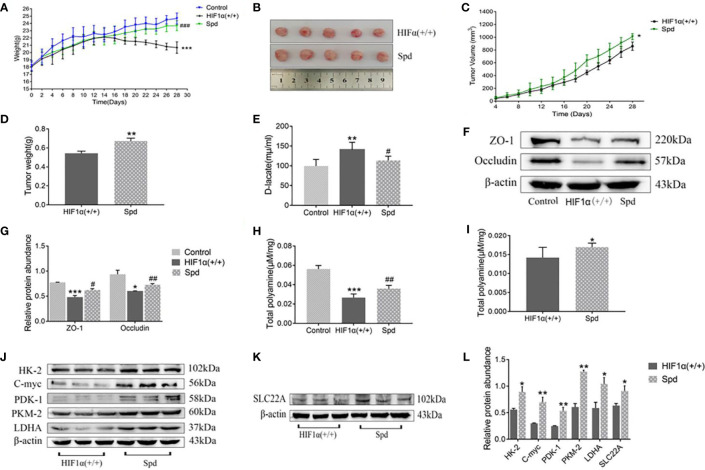
Effects of exogenous polyamine supplementation on tumor growth, intestinal mucosal permeability, and polyamines in tumor-bearing mice. **(A)** Body weight of tumor-bearing mice after exogenous polyamine supplementation. **(B)** Tumor tissue from tumor-bearing mice after exogenous polyamine supplementation. **(C)** Changes in tumor volume in tumor-bearing mice after exogenous polyamine supplementation. **(D)** Tumor mass in tumor-bearing mice after exogenous polyamine supplementation. **(E)** D-lactic acid value of tumor-bearing mice after supplementation with exogenous polyamines. **(F)** Expression of ZO-1 and occludin proteins in tumor-bearing mice after exogenous polyamine supplementation. **(G)** Western blot quantitative data of **(F)**. HIF1α (+/+) group, ^*^ *P* < 0.05, ^***^ *P* < 0.001 *vs*. control group; Spd group, ^#^ *P* < 0.05, ^##^ *P* < 0.01 *vs*. HIF1α (+/+) group. **(H)** Contents of polyamines in the intestinal mucosa of tumor-bearing mice after supplementation with exogenous polyamines. HIF1a (+/+) group, ***P < 0.001 vs. control group; Spd group, ##P < 0.01 vs. HIF1a (+/+) group. **(I)** Contents of polyamines in tumor tissues of tumor-bearing mice after exogenous polyamine supplementation.**(J)** The amount of glycolysis-related proteins in tumor-bearing mice after exogenous polyamine supplementation. **(K)** The amount of SLC22A protein in tumor-bearing mice after supplementation with exogenous polyamines. **(L)** Quantitative data of **(J)** and **(K)**. Spd group ^*^ *P*<0.05, ^**^ *P*<0.01 *vs*. HIF1α (+/+) group.

**Figure 7:**
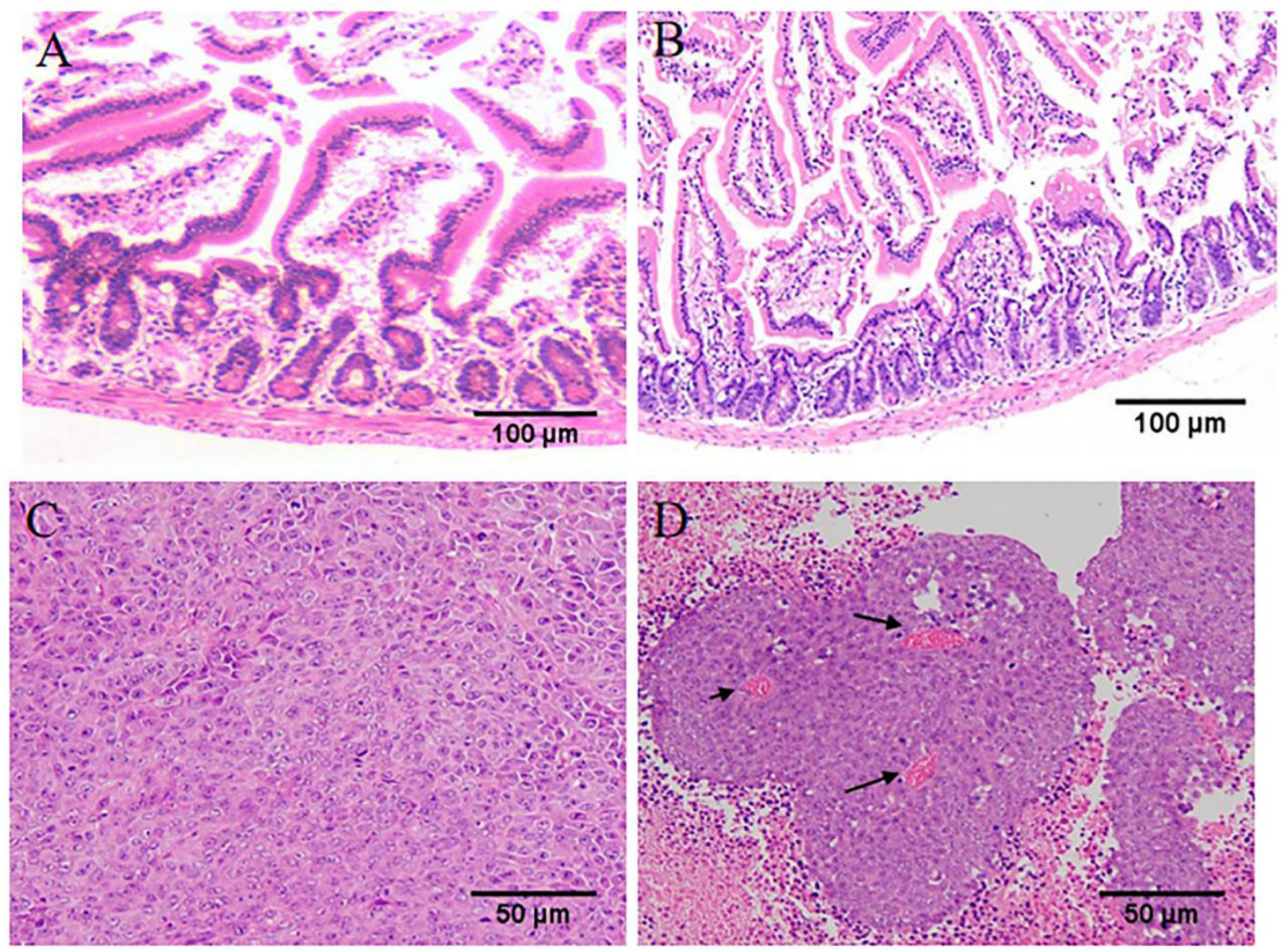
Supplementation of exogenous polyamines can meet the requirements of polyamines for tumor growth and avoid the predation of polyamines in the intestinal mucosa. **(A)** The structure of intestinal mucosa of tumor-bearing mice in HIF1α (+/+) group under light microscope. **(B)** Intestinal mucosal structure of tumor-bearing mice in Spd group. **(C)**. Tumor structure of tumor-bearing mice in HIF1α (+/+) group under light microscope. **(D)** Tumor structure of tumor-bearing mice in Spd group. (AB: ×100; CD: ×200).

During the gavage period, the long and short diameters of the tumors were measured using vernier calipers every other day. Increased tumor volume in the spermidine-administered HIF1α (+/+) group compared to the non-administered HIF1α (+/+) group (**Figure 6A**). After two weeks of gavage, it was found that the tumors in the spermidine group were larger (**Figure 6B**). As exhibited in **Figure 6C**, the weight of spermidine group was higher than that of the HIF1α (+/+) group after administration. As shown in **Figure 6D**, After the tumors were weighed, it was found that the tumor mass in the spermidine group was higher than that in the HIF1α (+/+) group. The results of the intestinal mucosal permeability test after spermidine administration are shown in **Figure 6E**. The serum D-lactic acid concentration in the HIF1α (+/+) group was higher than that in the spermidine group and higher than that in the normal group. Using WB method to detect the tight junction proteins in the HIF1α (+/+) group and the spermidine group (**Figure 6F**), we found that the protein expressions of ZO1 and Occludin were significantly up-regulated in the spermidine group (**Figure 6G**). The results of histopathological sections can be seen in **Figure 7**. After supplementation with exogenous polyamines, compared with the HIF1α (+/+) group, the spermidine group had significantly increased intestinal glands, closely arranged, and increased Paneth cells And the blood vessels in the tumor tissue increased significantly. Increased blood vessels is an important condition for tumor growth, infiltration and metastasis, and it is more conducive for tumors to absorb nutrients from the body for their own growth. Apparently, supplementation with exogenous polyamines protects the intestinal mucosa and promotes tumor growth in tumor-bearing mice. As shown in **Figures 6H, I**, the content of polyamines in the intestinal mucosa tissue of the normal group is the highest. The content of polyamines in intestinal mucosa tissue and tumor tissue in spermidine group was higher than that in HIF1α (+/+) group. Western blot was used to detect the expression levels of glycolysis-related proteins and SLC22A protein (**Figures 6J, K**), and the gray value analysis displayed that the expressions of these proteins were all up-regulated (**Figure 6L**). This indicates that supplementation of exogenous polyamines can promote tumor glycolysis, meet the needs of the tumor itself and avoid the predation of polyamines by the tumor on the intestinal mucosa.

## 4. Conclusion

In the malignant process of CRC, activation of HIF1 can promote tumor growth and glycolysis, and aggravate the damage of the intestinal mucosal barrier.As the degree of hypoxia in the tumor microenvironment deepens, HIF1α will inhibit the transcription of the c-Myc target gene ODC, and the reduction of polyamine synthesis in tumor tissue, the tumor will plunder polyamines from the intestinal mucosa to meet its own energy needs, ultimately leading to intestinal epithelial barrier dysfunction. Therefore, regulating tumor polyamine metabolism and inhibiting HIF1 pathway provide new ideas for the treatment of colorectal cancer.

## Data Availability Statement

The original contributions presented in the study are included in the article/supplementary material. Further inquiries can be directed to the corresponding author/s.

## Ethics Statement

This study was reviewed and approved by the Institutional Review Committee of the Animal Care and Use Committee of Guangdong Pharmaceutical University.

## Author contributions

YW, GW, and TL contributed to conception and design of the study. YW and JC organized the database. HH performed the statistical analysis. YW wrote the first draft of the manuscript. ZS, JC, HH, and XP wrote sections of the manuscript. All authors contributed to manuscript revision, read, and approved the submitted version.

## Funding

This work was financially supported by the National Natural Science Foundation of China (NO.81873225).

## Conflict of Interest

The authors declare that the research was conducted in the absence of any commercial or financial relationships that could be construed as a potential conflict of interest.

## Publisher’s Note

All claims expressed in this article are solely those of the authors and do not necessarily represent those of their affiliated organizations, or those of the publisher, the editors and the reviewers. Any product that may be evaluated in this article, or claim that may be made by its manufacturer, is not guaranteed or endorsed by the publisher.
